# The 100 most cited articles in androgenetic alopecia: A bibliometric analysis

**DOI:** 10.1097/MD.0000000000041881

**Published:** 2025-03-21

**Authors:** Kinan Sawar, Louie Mekani, Alexander Kallabat, Dylan Kato, Geoffrey A. Potts

**Affiliations:** a Department of Surgery, Section of Plastic Surgery, University of Michigan Medical School, Ann Arbor, MI; b Wayne State University School of Medicine, Detroit, MI; c Department of Dermatology, Wayne State University, Detroit, MI.

**Keywords:** androgenetic alopecia, bibliometric analysis

## Abstract

Androgenetic alopecia (AGA) is a condition with a rapidly evolving research landscape. The expanding volume of AGA research necessitates synthesis to identify trends and gaps. Bibliometric analysis can be used to quantify the characteristics of a body of literature, and this technique has not been previously applied to AGA. We aimed to evaluate the bibliometric characteristics of the top 100 most cited AGA articles over the past 50 years. This includes the analysis of contributing authorship, institutional affiliation, journal representation, publication years, citation counts, country productivity, and funding sources, among other characteristics. We used the Web of Science to identify and characterize the 100 most cited AGA publications between 1975 and 2024. A secondary analysis was performed to identify and characterize the top 20 most cited AGA articles from 2020 to 2024. The top 100 articles amassed 24,289 citations. Authors from the United States contributed the most articles (n = 56). The University of Pennsylvania contributed the most articles (n = 11). Dr Elise A Olsen and Dr George Cotsarelis were the most frequent contributors. The *Journal of the American Academy of Dermatology* published the most number of articles. From 1975 to 2024, most studies primarily discussed pathophysiology (45%) and treatment (35%). However, from 2020 to 2024, 75% of the top articles focused on the treatment options. This bibliometric analysis provides an overview of influential AGA research over the last 50 years, highlighting shifting trends toward treatment optimization and emerging therapies.

## 1. Introduction

Androgenetic alopecia (AGA), a condition affecting millions of people worldwide, is recognized as the most common cause of non-scarring alopecia.^[1]^ Research related to AGA has significantly evolved over the last few decades as an understanding of its pathophysiology has led to the development of more effective treatment options. The development of pharmacological, device-based, and surgical treatment options has collectively contributed to the management of AGA. Established treatment options, including 5-α reductase (5-AR) inhibitors, minoxidil, platelet-rich plasma (PRP), low-level laser therapy (LLLT), and hair transplantation, among other options, have undergone extensive research and have been used in clinical practice for years.^[1]^ New AGA treatment options are actively being investigated, including pyrilutamide (originally named KX-826), GT20029, and exosome therapy.^[2–4]^ Given the increasing volume of research related to AGA, there is a growing need to synthesize the expansive body of literature on AGA to identify trends in research output, influential publications, and potential gaps in research.

Bibliometric analysis is a scientific methodology that is used to quantify the characteristics of a body of literature.^[5]^ Characteristics that are evaluated include measures such as author H-index, author and article citation counts, and institutional funding sources. Quantifying these variables allows researchers to quickly identify influential studies, prominent authors, and emerging trends within the field to guide individual research efforts, identify opportunities for collaboration, and determine possible sources of funding.^[5]^ Bibliometric analysis has been used to evaluate many disciplines within dermatology but has yet to be applied to AGA literature.^[6–9]^ The aim of this study was to evaluate the characteristics of the top 100 cited articles on the topic of androgenetic alopecia in the last 50 years. This analysis includes an evaluation of the contributing authors, affiliated institutions, journals, years of publication, citation frequency, countries of origin, and funding sources of the top-cited articles related to AGA.

## 2. Methods

The Web of Science platform was used to identify the 100 most cited publications on AGA in the last 50 years (January 1, 1975 to June 30, 2024). Articles were analyzed for title, authorship, institution, journal, year, citation count, publishing country, funding status, citation index, Altmetric attention score (AAS), impact factor, Eigenfactor score, and article influence score. All articles were categorized into AGA-relevant subtopics: pathophysiology, diagnosis, treatment, side effects, psychosocial effects, and an “other” category for those that did not fit the listed categories.

The search was conducted on July 1, 2024. The exact search methodology used to identify the top 100 most cited research articles on AGA published between 1975 and 2024 utilized is provided: “androgenetic alopecia” OR “androgenic alopecia” OR “hair loss” OR “pattern baldness” OR “common baldness” OR (“minoxidil” OR “finasteride” OR “dutasteride” OR “GT20029” OR “pyrilutamide” OR “rosemary” OR “saw palmetto” OR microneedle* OR “red light” OR “low level laser therapy” OR “low-level laser therapy” OR “LLLT” OR “PRP” OR “platelet-rich plasma” OR “dihydrotestosterone” OR “DHT” OR androgen* OR “5-α reductase” OR “5α reductase”) AND (hair* OR alopecia* OR bald*). The search resulted in 14,867 articles. These articles were then ordered by citation number from highest to lowest, and the full text was manually screened to determine whether each article was investigating AGA in any context, including but not limited to the pathophysiology, diagnosis, treatment, treatment side effects, and psychosocial effects related to AGA. Articles discussing the general physiology of hair growth or the general pathophysiology of hair loss were included as long as they also discussed AGA. Articles in which AGA was mentioned as a diagnosis secondary to an associated medical condition were excluded from analysis. To assess insights regarding recent AGA related publications, the authors conducted an identical subsequent analysis that identified and characterized the top 20 most cited articles from the last 5 years (January 1, 2020 to June 30, 2024) using the same search parameters. The search resulted in 4804 articles. Previous bibliometric analyses evaluating other dermatology topics have implemented similar analysis.^[6]^ This study used public data that did not involve human subjects, and therefore did not require Institutional Review Board approval.

## 3. Results

We identified the top 100 cited articles among the 14,106 articles that met the initial search criteria (Table S1, Supplemental Digital Content, http://links.lww.com/MD/O566). The top 100 articles ranged between 140 and 1019 citations per article (mean of 243 times, median of 186 times) for a combined 24,289 total number of citations. The average number of citations per year was also calculated, ranging between 4.2 and 67.9 (mean of 13.6, median of 10.4). Most articles were published in 2002 and 2004 (Figures S1 and S2, Supplemental Digital Content, http://links.lww.com/MD/O567). The most cited article over the last 50 years was “Capturing and profiling adult hair follicle stem cells,” authored by Rebecca J. Morris, Yaping Liu, Lee Marles, Zaixin Yang, Carol Trempus, Shulan Li, Jamie S. Lin, Janet A. Sawicki, and George Cotsarelis (Table 1). It was published in *Nature Biotechnology* in 2004 and has been cited 1019 times and averages 48.52 citations per year.

**Table 1:** List of top 10 most cited AGA articles.

Rank	Title	Authors	Journal	Publication year	Total citations	Average per year	Altmetric score
1	Capturing and profiling adult hair follicle stem cells	Morris, RJ; Liu, YP; Marles, L; Yang, ZX; Trempus, C; Li, SL; Lin, JS; Sawicki, JA; Cotsarelis, G	*Nature Biotechnology*	2004	1019	48.5	15
2	The biology of hair follicles	Paus, R; Cotsarelis, G	*New England Journal of Medicine*	1999	898	34.5	63
3	The nuts and bolts of low-level laser (light) therapy	Chung, Hoon; Dai, Tianhong; Sharma, Sulbha K; Huang, Ying-Ying; Carroll, James D; Hamblin, Michael R	*Annals of Biomedical Engineering*	2012	883	67.9	168
4	Wnt-dependent De Novo hair follicle regeneration in adult mouse skin after wounding	Ito, Mayumi; Yang, Zaixin; Andl, Thomas; Cui, Chunhua; Kim, Noori; Millar, Sarah E; Cotsarelis, George	*Nature*	2007	796	44.2	36
5	Molecular mechanisms regulating hair follicle development	Millar, SE	*Journal of Investigative Dermatology*	2002	733	31.9	16
6	Tissue distribution and ontogeny of steroid 5-alpha-reductase isozyme expression	Thigpen, AE; Silver, RI; Guileyardo, JM; Casey, ML; McConnell, JD; Russell, DW	*Journal of Clinical Investigation*	1993	601	18.8	15
7	Androgen excess in women: experience with over 1000 consecutive patients	Azziz, R; Sanchez, LA; Knochenhauer, ES; Moran, C; Lazenby, J; Stephens, KC; Taylor, K; Boots, LR	*Journal of Clinical Endocrinology & Metabolism*	2004	544	25.9	12
8	Male pattern baldness—classification and incidence	Norwood, OT	*Southern Medical Journal*	1975	518	10.4	23
9	Classification of types of androgenetic alopecia (common baldness) occurring in female sex	Ludwig, E	*British Journal of Dermatology*	1977	504	10.5	13
10	Finasteride in the treatment of men with androgenetic alopecia	Kaufman, KD; Olsen, EA; Whiting, D; Savin, R; DeVillez, R; Bergfeld, W; Price, VH; Van Neste, D; Roberts, JL; Hordinsky, M; Shapiro, J; Binkowitz, B; Gormley, GJ	*Journal of the American Academy of Dermatology*	1998	465	17.2	164

AGA = androgenetic alopecia.

A total of 463 different authors contributed to the publication of the top 100 articles (Table 2). The most common contributors to this list were Dr Elise A Olsen (n = 7) and Dr George Cotsarelis (n = 6). The primary author contributions came from 17 different countries (Table S2, Supplemental Digital Content, http://links.lww.com/MD/O568). The United States contributed the highest number of articles (n = 56), followed by England (n = 14) and Germany (n = 12). Two hundred twenty-eight different institutions contributed to the top 100 articles (Table S3, Supplemental Digital Content, http://links.lww.com/MD/O569), with the University of Pennsylvania contributing the most (n = 11), followed by Duke University (n = 7), University of California—System (n = 7), and University of Texas—System (n = 7). Thirty different funding agencies contributed to these articles, with the National Institutes of Health and the United States of America being the most common contributors (n = 14) (Table S4, Supplemental Digital Content, http://links.lww.com/MD/O570).

**Table 2:** Author contribution to the top 100 AGA articles.

Rank	Author*	# of Top 100 Publications	# of Citations	H-index
1	Olsen EA	7	8467	48
2	Cotsarelis G	6	17,178	49
3	Millar SE	5	11,925	49
4	Price VH	5	5180	38
5	Hordinsky M	4	6812	39
6	Messenger AG	4	6773	48
7	Paus R	4	41,089	107
8	Thiboutot D	4	6665	44
9	Cash TF	3	10,600	56
10	Foitzik K	3	3437	21
11	Garcovich S	3	2299	27
12	Gentile P	3	5078	46
13	Roberts JL	3	11,102	55
14	Tosti A	3	21,217	63
15	Whiting DA	3	7792	45
16	Yang ZX	3	1447	7

AGA = androgenetic alopecia.

* 447 additional authors contributed to ≤ 2 publications among the top 100 most cited articles and were not included in this table.

**Figure 1.**
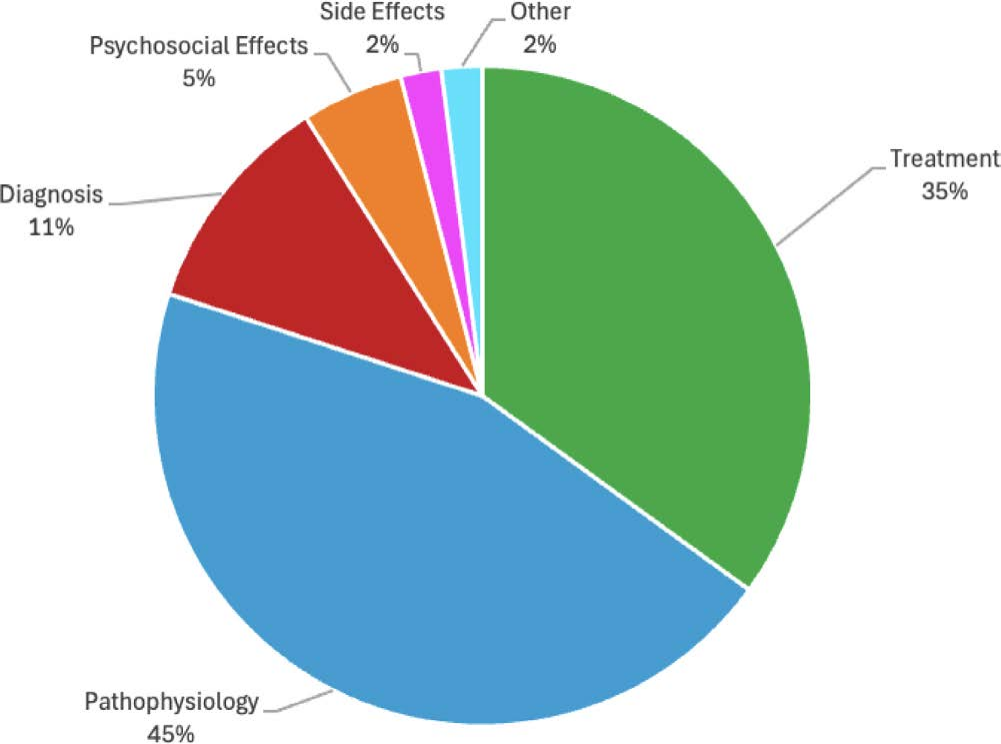
Pie chart of study categories represented in the top 100 most cited AGA publications from 1975 to 2024. AGA = androgenetic alopecia.

The article “The nuts and bolts of low-level laser (light) therapy” by Chung et al had the highest number of average citations per year, with an average of 67.92. Suchonwanit et al 2019 article “Minoxidil and its use in hair disorders: a review” had the highest AAS of 618 (Table S1, Supplemental Digital Content, http://links.lww.com/MD/O566). The journals most frequently represented in the top 100 list included *Journal of the American Academy of Dermatology* (n = 16) and *Journal of Investigative Dermatology* (n = 11). *New England Journal of Medicine* had the highest impact factor (96.2) and the highest article influence score (41.530) (Table S5, Supplemental Digital Content, http://links.lww.com/MD/O571). The largest category of studies from the top 100 list primarily discussed the pathophysiology of AGA (45%), followed by treatment (35%), diagnosis (11%), side effects (5%), psychosocial effects (2%), and all other topics (2%) (Fig. 1).

**Figure 2.**
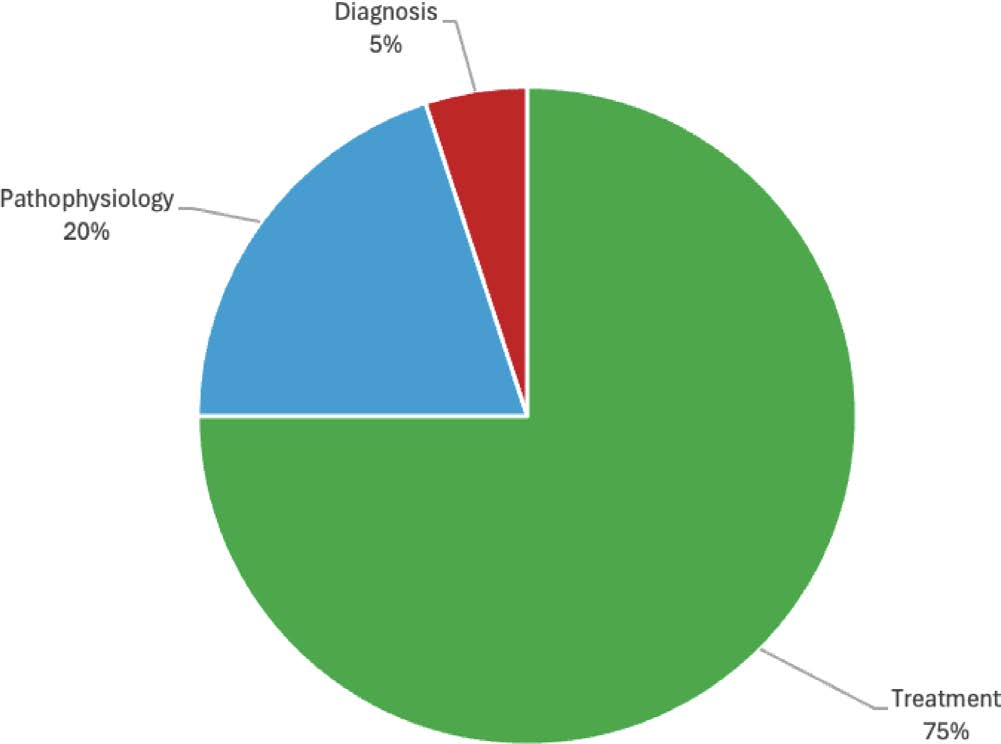
Pie chart of study categories represented in the top 20 most cited AGA publications from 2020 to 2024. AGA = androgenetic alopecia.

The secondary analysis discussing the 20 most highly cited articles related to AGA between 2020 and 2024 revealed that the article titled “Targeting Wnt/β-catenin pathway for developing therapies for hair loss” published in 2020 by Bu Young Choi in *International Journal of Molecular Sciences* was the most highly cited during this time period (Table S6, Supplemental Digital Content, http://links.lww.com/MD/O572). This article was cited 111 times, averaging 28 citations per year. *Journal of the American Academy of Dermatology* was the most represented journal during this period. The analysis of article categories showed that 75% of studies primarily discussed treatment, followed by pathophysiology (20%) and diagnosis (5%) (Fig. 2). No studies in this list primarily focused on the side effects or psychosocial effects related to AGA.

## 4. Discussion

This bibliometric study provides an analysis of the characteristics of highly cited AGA research articles, such as contributing authors, article citation count, AAS, and author H-index. As of July 2024, the top 100 articles were collectively cited 24,289 times. The top-cited article, “Capturing and profiling adult hair follicle stem cells,” was written by Morris et al in 2004. Authors affiliated with the University of Pennsylvania contributed the most articles. The top 2 contributing authors were Dr Elise A Olsen from Duke University Medical Center and Dr George Cotsarelis from University of Pennsylvania School of Medicine. *Journal of the American Academy of Dermatology* published the highest number of articles on this list. While first authors from the United States contributed to the greatest number of articles, first authors from 17 different countries contributed to articles in the top 100 list, underscoring the global interest in AGA research.

The oldest article included in the top 100 list is titled “Male pattern baldness—classification and incidence” by Norwood, which was published in 1975.^[10]^ This was the first ever article to describe a classification system that could be used to assess the progression of AGA in male patients. The Norwood–Hamilton scale is still commonly used to assess the progression of AGA in male patients. A newer AGA classification system, the basic and specific classification, was developed decades later in 2007 and is more versatile because it characterizes AGA in both male and female patients but has yet to gain widespread popularity for use in the assessment of AGA progression.^[11]^ The PRECISE scale is another newly developed AGA classification system and has been increasingly adopted by hair transplant surgeons as a quantitative tool to classify male AGA patients during preoperative planning.^[11]^ However, the PRECISE scale, another new classification system for AGA, is gaining traction specifically among hair transplant surgeons as a quantitative tool to classify male AGA patients during preoperative planning.^[12]^ These new classification systems represent an advancement from the qualitative categories of the Hamilton-Norwood scale. Over the last 50 years, the focus of AGA research has evolved substantially. The earliest studies characterized hormonal and genetic factors contributing to AGA.^[13–16]^ However, advancement in our understanding of both the pathophysiology and treatment of AGA has contributed to an increase in focused research subtopics such as Wnt signaling,^[17–19]^ 5-AR inhibitor use,^[20–23]^ and PRP.^[19,24–27]^ The first clinical trial demonstrating the efficacy of topical finasteride was completed in 1997.^[12]^ Since this initial publication, many studies have evaluated the use of 5-AR inhibitors in the treatment of AGA.^[16,21,23,28–39]^

Current AGA research has built upon our improved understanding of AGA pathophysiology to develop better pharmacologic, device-based, and surgical treatments. Topical minoxidil and LLLT are currently the only FDA approved treatments for female AGA, while these treatments in addition to oral finasteride are approved for male AGA.^[40]^ Newer, non-FDA approved treatments such as dutasteride, GT20029, exosome therapy, and pyrilutamide have become more heavily researched in recent years.^[2,3,34,41]^ These newer treatments are promising. For example, dutasteride has demonstrated a greater increase in hair counts per square centimeter compared to oral finasteride while maintaining a similar rate of side effects.^[34,42]^ Dutasteride’s clinical superiority to finasteride has been associated with its improved ability to reduce hair follicle DHT compared to finasteride.^[43]^ Despite dutasteride’s success in the treatment of AGA, only one of the top 100 articles evaluated long-term AGA outcomes with its use.^[34]^ In contrast, multiple studies included in this list have assessed long-term AGA outcomes with finasteride monotherapy.^[32,34,35]^

In addition to dutasteride, other emerging treatments for AGA, such as exosome therapy, pyrilutamide, and GT20029, are beginning to be researched. Exosome therapy is promising considering that preclinical studies have revealed that exosomes contribute to hair follicle regeneration via the delivery of proteins and mRNA, which have been shown to activate essential signaling pathways involved in hair growth such as the Wnt/β-catenin pathway.^[41]^ Pyrilutamide and GT20029 are also promising for the treatment of AGA as these treatment options are both currently being evaluated in clinical trials.^[2,3]^ Pyrilutamide functions as a direct androgen receptor antagonist whereas GT20029 facilitates the degradation of the androgen receptor using ubiquitin-mediated proteolysis.^[44]^ Unfortunately, none of the top 100 articles discuss exosome therapy, pyrilutamide, or GT20029. However, 2 of the articles in our analysis from 2020 to 2024 discuss the use of exosomes for hair growth, indicating that research efforts are beginning to target these new treatment modalities.

In addition to the research trends identified in our top-cited articles, it is important to consider emerging evidence that suggests a potential causational link between androgenetic alopecia and COVID-19 severity.^[45]^ Studies have suggested that AGA may predispose patients to more severe SARS-CoV-2 infection via an androgen mediated pathway.^[46]^ Specifically, transmembrane protease serine 2 is activated by androgens and has been posited to help mediate the entrance of COVID-19 into cells through the ACE-2 receptor. These findings warrant further investigation and may have important implications for future research priorities related to AGA.

Our analysis of article categories from 1975–2024 to 2020–2024 demonstrates that there is an increased focus on research of treatment options for AGA and less focus on elucidating the pathophysiology of AGA. Given the success of a variety of treatment options, many studies have begun to comparatively evaluate these different treatment options, both as monotherapy and combination therapy. Additionally, our analysis shows an increase in the number of publications evaluating the impact of an individual treatment’s dosage on clinical outcomes. Ultimately, this article highlights the shifting landscape of AGA research, emphasizing a growing focus on treatment optimization while also pointing to emerging therapies for AGA treatment.

## 5. Conclusions

This is the first article to identify the most highly cited papers in the field of AGA. Healthcare practitioners, researchers, and policymakers can use curated landmark articles to explore the field of AGA and understand the changing landscape of AGA research.

## Author contributions

**Conceptualization:** Kinan Sawar, Alexander Kallabat, Dylan Kato, Geoffrey A. Potts.

**Data curation:** Kinan Sawar, Louie Mekani.

**Formal analysis:** Kinan Sawar, Louie Mekani.

**Investigation:** Kinan Sawar.

**Methodology:** Kinan Sawar.

**Project administration:** Kinan Sawar, Geoffrey A. Potts.

**Resources:** Kinan Sawar.

**Software:** Kinan Sawar.

**Supervision:** Kinan Sawar, Geoffrey A. Potts.

**Validation:** Kinan Sawar, Louie Mekani, Geoffrey A. Potts.

**Visualization:** Kinan Sawar, Louie Mekani.

**Writing—original draft:** Kinan Sawar, Louie Mekani, Alexander Kallabat, Dylan Kato.

**Writing—review & editing:** Kinan Sawar, Louie Mekani, Alexander Kallabat, Dylan Kato, Geoffrey A. Potts.

## Supplementary Material

SUPPLEMENTARY MATERIAL

## References

[1] Ntshingila S Oputu O Arowolo AT Khumalo NP. Androgenetic alopecia: an update. JAAD Int. 2023;13:150–8. 37823040 10.1016/j.jdin.2023.07.005 PMC10562178

[2] Zhang J Yang Q. Phase III study of KX-826 with adult male patients with AGA. Available at: https://www.clinicaltrials.gov/study/NCT06126965?cond=kx-826&rank=2 [access date August 12, 2024].

[3] Overcash SJ Bertoch TM. To evaluate the safety, tolerability and PK of GT20029 gel and solution in healthy subjects. Available at: https://www.clinicaltrials.gov/study/NCT06468579?cond=gt20029&rank=2 [access date August 12, 2024].

[4] Ersan M. Exosome treatment in androgenetic alopecia. Available at: https://www.clinicaltrials.gov/study/NCT06539273?cond=Exosome&rank=3 [access date August 12, 2024]. 10.1007/s00266-024-04332-3 PMC11588828 39174804

[5] Ellegaard O Wallin JA. The bibliometric analysis of scholarly production: how great is the impact? Scientometrics. 2015;105:1809–31. 26594073 10.1007/s11192-015-1645-z PMC4643120

[6] Pakhchanian H Aizman L Raiker R Ng E. Top 100 most cited articles in MOHS micrographic surgery: a bibliometric analysis. Dermatol Surg. 2024;50:137–43. 37994504 10.1097/DSS.0000000000004023

[7] Li J Wang L Yin S. Emerging trends and hotspots of the itch research: a bibliometric and visualized analysis. CNS Neurosci Ther. 2024;30:e14514. 37902196 10.1111/cns.14514 PMC11017449

[8] Jia L Guo R Ying J Xiong J Jiang H. A bibliometric and visualized research on global trends of scar, 2011–2021. Burns. 2023;49:1557–65. 37217380 10.1016/j.burns.2023.04.010

[9] Molina-García M Granger C Trullàs C Puig S. Exposome and skin: part 1. Bibliometric analysis and review of the impact of exposome approaches on dermatology. Dermatol Ther (Heidelb). 2022;12:345–59. 35112325 10.1007/s13555-021-00680-z PMC8850514

[10] Norwood OT. Male pattern baldness: classification and incidence. South Med J. 1975;68:1359–65. 1188424 10.1097/00007611-197511000-00009

[11] Lee WS Ro BI Hong SP. A new classification of pattern hair loss that is universal for men and women: basic and specific (BASP) classification. J Am Acad Dermatol. 2007;57:37–46. 17467851 10.1016/j.jaad.2006.12.029

[12] Pittella F Castro CG Trivellini R. PRECISE Scale: a quantitative classification for androgenetic alopecia and its application to hair transplantation. Aesthetic Plast Surg. 2024;48:775–84. 37365308 10.1007/s00266-023-03462-4 PMC10980655

[13] Lookingbill DP Demers LM Wang C Leung A Rittmaster RS Santen RJ. Clinical and biochemical parameters of androgen action in normal healthy Caucasian versus Chinese subjects. J Clin Endocrinol Metab. 1991;72:1242–8. 1827450 10.1210/jcem-72-6-1242

[14] Choudhry R Hodgins MB Van der kwast TH Brinkmann AO Boersma WJ. Localization of androgen receptors in human skin by immunohistochemistry: implications for the hormonal-regulation of hair-growth, sebaceous glands and sweat glands. J Endocrinol. 1992;133:467–75. 1613448 10.1677/joe.0.1330467

[15] Carey AH Chan KL Short F White D Williamson R Franks S. Evidence for a single gene effect causing polycystic ovaries and male pattern baldness. Clin Endocrinol (Oxf). 1993;38:653–8. 8334753 10.1111/j.1365-2265.1993.tb02150.x

[16] Thigpen AE Silver RI Guileyardo JM Casey ML McConnell JD Russell DW. Tissue distribution and ontogeny of steroid 5-alpha-reductase isozyme expression. J Clin Invest. 1993;92:903–10. 7688765 10.1172/JCI116665 PMC294929

[17] Castilho RM Squarize CH Chodosh LA Williams BO Gutkind JS. mTOR mediates Wnt-induced epidermal stem cell exhaustion and aging. Cell Stem Cell. 2009;5:279–89. 19733540 10.1016/j.stem.2009.06.017 PMC2939833

[18] Huang P Yan R Zhang X Wang L Ke X Qu Y. Activating Wnt/beta-catenin signaling pathway for disease therapy: challenges and opportunities. Pharmacol Ther. 2019;196:79–90. 30468742 10.1016/j.pharmthera.2018.11.008

[19] Gentile P Garcovich S. Advances in regenerative stem cell therapy in androgenic alopecia and hair loss: Wnt pathway, growth-factor, and mesenchymal stem cell signaling impact analysis on cell growth and hair follicle development. Cells. 2019;8:466. 31100937 10.3390/cells8050466 PMC6562814

[20] Blumeyer A Tosti A Messenger A. Evidence-based (S3) guideline for the treatment of androgenetic alopecia in women and in men. J Dtsch Dermatol Ges. 2011;9(Suppl 6):S1–57. 10.1111/j.1610-0379.2011.07802.x 21980982

[21] Azzouni F Godoy A Li Y Mohler J. The 5 alpha-reductase isozyme family: a review of basic biology and their role in human diseases. Adv Urol. 2012;2012:530121. 22235201 10.1155/2012/530121 PMC3253436

[22] Lolli F Pallotti F Rossi A. Androgenetic alopecia: a review. Endocrine. 2017;57:9–17. 28349362 10.1007/s12020-017-1280-y

[23] Adil A Godwin M. The effectiveness of treatments for androgenetic alopecia: a systematic review and meta-analysis. J Am Acad Dermatol. 2017;77:136–41.e5. 28396101 10.1016/j.jaad.2017.02.054

[24] Li ZJ Choi HI Choi DK. Autologous platelet-rich plasma: a potential therapeutic tool for promoting hair growth. Dermatol Surg. 2012;38:1040–6. 22455565 10.1111/j.1524-4725.2012.02394.x

[25] Gentile P Garcovich S Bielli A Scioli MG Orlandi A Cervelli V. The effect of platelet-rich plasma in hair regrowth: a randomized placebo-controlled trial. Stem Cells Transl. Med. 2015;4:1317–23. 26400925 10.5966/sctm.2015-0107 PMC4622412

[26] Cervelli V Garcovich S Bielli A. The effect of autologous activated platelet rich plasma (AA-PRP) injection on pattern hair loss: clinical and histomorphometric evaluation. Biomed Res Int. 2014;2014:760709. 24883322 10.1155/2014/760709 PMC4032742

[27] Alves R Grimalt R. A review of platelet-rich plasma: history, biology, mechanism of action, and classification. Skin Appendage Disord. 2018;4:18–24. 29457008 10.1159/000477353 PMC5806188

[28] Dallob AL Sadick NS Unger W. The effect of finasteride, a 5 alpha-reductase inhibitor, on scalp skin testosterone and dihydrotestosterone concentrations in patients with male pattern baldness. J Clin Endocrinol Metab. 1994;79:703–6. 8077349 10.1210/jcem.79.3.8077349

[29] Hiipakka RA Zhang HZ Dai W Dai Q Liao S. Structure–activity relationships for inhibition of human 5alpha-reductases by polyphenols. Biochem Pharmacol. 2002;63:1165–76. 11931850 10.1016/s0006-2952(02)00848-1

[30] Imperato-McGinley J Zhu YS. Androgens and male physiology the syndrome of 5alpha-reductase-2 deficiency. Mol Cell Endocrinol. 2002;198:51–9. 12573814 10.1016/s0303-7207(02)00368-4

[31] Irwig MS Kolukula S. Persistent sexual side effects of finasteride for male pattern hair loss. J Sex Med. 2011;8:1747–53. 21418145 10.1111/j.1743-6109.2011.02255.x

[32] Kaufman KD Olsen EA Whiting D. Finasteride in the treatment of men with androgenetic alopecia. Finasteride Male Pattern Hair Loss Study Group. J Am Acad Dermatol. 1998;39(4 Pt 1):578–89. 9777765 10.1016/s0190-9622(98)70007-6

[33] Leyden J Dunlap F Miller B. Finasteride in the treatment of men with frontal male pattern hair loss. J Am Acad Dermatol. 1999;40(6 Pt 1):930–7. 10365924 10.1016/s0190-9622(99)70081-2

[34] Olsen EA Hordinsky M Whiting D. The importance of dual 5alpha-reductase inhibition in the treatment of male pattern hair loss: results of a randomized placebo-controlled study of dutasteride versus finasteride. J Am Acad Dermatol. 2006;55:1014–23. 17110217 10.1016/j.jaad.2006.05.007

[35] Price VH Roberts JL Hordinsky M. Lack of efficacy of finasteride in postmenopausal women with androgenetic alopecia. J Am Acad Dermatol. 2000;43(5 Pt 1):768–76. 11050579 10.1067/mjd.2000.107953

[36] Sawaya ME Price VH. Different levels of 5alpha-reductase type I and II, aromatase, and androgen receptor in hair follicles of women and men with androgenetic alopecia. J Invest Dermatol. 1997;109:296–300. 9284093 10.1111/1523-1747.ep12335779

[37] Thiboutot D Harris G Iles V Cimis G Gilliland K Hagari S. Activity of the type 1 5 alpha-reductase exhibits regional differences in isolated sebaceous glands and whole skin. J Invest Dermatol. 1995;105:209–14. 7636302 10.1111/1523-1747.ep12317162

[38] Traish AM Hassani J Guay AT Zitzmann M Hansen ML. Adverse side effects of 5alpha-reductase inhibitors therapy: persistent diminished libido and erectile dysfunction and depression in a subset of patients. J Sex Med. 2011;8:872–84. 21176115 10.1111/j.1743-6109.2010.02157.x

[39] Trueb RM. Molecular mechanisms of androgenetic alopecia. Exp Gerontol. 2002;37:981–90. 12213548 10.1016/s0531-5565(02)00093-1

[40] Nestor MS Ablon G Gade A Han H Fischer DL. Treatment options for androgenetic alopecia: efficacy, side effects, compliance, financial considerations, and ethics. J Cosmet Dermatol. 2021;20:3759–81. 34741573 10.1111/jocd.14537 PMC9298335

[41] Kost Y Muskat A Mhaimeed N Nazarian RS Kobets K. Exosome therapy in hair regeneration: a literature review of the evidence, challenges, and future opportunities. J Cosmet Dermatol. 2022;21:3226–31. 35441799 10.1111/jocd.15008

[42] Shanshanwal SJ Dhurat RS. Superiority of dutasteride over finasteride in hair regrowth and reversal of miniaturization in men with androgenetic alopecia: a randomized controlled open-label, evaluator-blinded study. Indian J Dermatol Venereol Leprol. 2017;83:47–54. 27549867 10.4103/0378-6323.188652

[43] Hobo Y Nishikawa J Taniguchi Asai N. Evaluation of the therapeutic effects of AGA drugs by measuring finasteride, dutasteride, and dihydrotestosterone in hair. Clin Chim Acta. 2023;547:117456. 37385468 10.1016/j.cca.2023.117456

[44] Saceda-Corralo D Dominguez-Santas M Vano-Galvan S Grimalt R. What’s new in therapy for male androgenetic alopecia? Am J Clin Dermatol. 2023;24:15–24. 36169916 10.1007/s40257-022-00730-y

[45] Moravvej H Pourani MR Baghani M Abdollahimajd F. Androgenetic alopecia and COVID-19: a review of the hypothetical role of androgens. Dermatol Ther. 2021;34:e15004. 34033224 10.1111/dth.15004 PMC8209856

[46] Asselta R Paraboschi EM Mantovani A Duga S. ACE2 and TMPRSS2 variants and expression as candidates to sex and country differences in COVID-19 severity in Italy. Aging (Albany, NY). 2020;12:10087–98. 32501810 10.18632/aging.103415 PMC7346072

