# Supplementary material for: The 100 most cited articles in androgenetic alopecia: A bibliometric analysis
Source: Medicine (Baltimore). 2025 Mar 21;104(12):e41881. doi: 10.1097/MD.0000000000041881 (PMC11936583; doi:10.1097/MD.0000000000041881)
Supplement: SUPPLEMENTARY MATERIAL [file medi-104-e41881-s001.docx]

| **Rank** | **Title** | **Authors** | **Journal** | **Publication Year** | **Total Citations** | **Average Per Year** | **Altmetric Score** |
| --- | --- | --- | --- | --- | --- | --- | --- |
| 1 | Capturing and Profiling Adult Hair Follicle Stem Cells | Morris, RJ; Liu, YP; Marles, L; Yang, ZX; Trempus, C; Li, SL; Lin, JS; Sawicki, JA; Cotsarelis, G | Nature Biotechnology | 2004 | 1,019 | 48.5 | 15 |
| 2 | The Biology of Hair Follicles | Paus, R; Cotsarelis, G | New England Journal of Medicine | 1999 | 898 | 34.5 | 63 |
| 3 | The Nuts and Bolts of Low-Level Laser (Light) Therapy | Chung, Hoon; Dai, Tianhong; Sharma, Sulbha K.; Huang, Ying-Ying; Carroll, James D.; Hamblin, Michael R. | Annals of Biomedical Engineering | 2012 | 883 | 67.9 | 168 |
| 4 | Wnt-Dependent De Novo Hair Follicle Regeneration in Adult Mouse Skin After Wounding | Ito, Mayumi; Yang, Zaixin; Andl, Thomas; Cui, Chunhua; Kim, Noori; Millar, Sarah E.; Cotsarelis, George | Nature | 2007 | 796 | 44.2 | 36 |
| 5 | Molecular Mechanisms Regulating Hair Follicle Development | Millar, SE | Journal of Investigative Dermatology | 2002 | 733 | 31.9 | 16 |
| 6 | Tissue Distribution and Ontogeny of Steroid 5-Alpha-Reductase Isozyme Expression | Thigpen, AE; Silver, RI; Guileyardo, JM; Casey, ML; McConnell, JD; Russell, DW | Journal of Clinical Investigation | 1993 | 601 | 18.8 | 15 |
| 7 | Androgen Excess in Women: Experience with Over 1000 Consecutive Patients | Azziz, R; Sanchez, LA; Knochenhauer, ES; Moran, C; Lazenby, J; Stephens, KC; Taylor, K; Boots, LR | Journal of Clinical Endocrinology & Metabolism | 2004 | 544 | 25.9 | 12 |
| 8 | Male Pattern Baldness - Classification and Incidence | Norwood, OT | Southern Medical Journal | 1975 | 518 | 10.4 | 23 |
| 9 | Classification of Types of Androgenetic Alopecia (Common Baldness) Occurring in Female Sex | Ludwig, E | British Journal of Dermatology | 1977 | 504 | 10.5 | 13 |
| 10 | Finasteride in the Treatment of Men with Androgenetic Alopecia | Kaufman, KD; Olsen, EA; Whiting, D; Savin, R; DeVillez, R; Bergfeld, W; Price, VH; Van Neste, D; Roberts, JL; Hordinsky, M; Shapiro, J; Binkowitz, B; Gormley, GJ | Journal of the American Academy of Dermatology | 1998 | 465 | 17.2 | 164 |
| 11 | Polycystic Ovaries and Premature Male Pattern Baldness are Associated with One Allele of the Steroid-Metabolism Gene CYP17 | Carey, AH; Waterworth, D; Patel, K; White, D; Little, J; Novelli, P; Franks, S; Williamson, R | Human Molecular Genetics | 1994 | 380 | 12.3 | 7 |
| 12 | Minoxidil: Mechanisms of Action on Hair Growth | Messenger, AG; Rundegren, J | British Journal of Dermatology | 2004 | 364 | 17.3 | 69 |
| 13 | Different Levels of 5 Alpha-Reductase Type I and II, Aromatase, and  Androgen Receptor in Hair Follicles of Women and Men with Androgenetic Alopecia | Sawaya, ME; Price, VH | Journal of Investigative Dermatology | 1997 | 335 | 12.0 | 7 |
| 14 | mTOR Mediates Wnt-Induced Epidermal Stem Cell Exhaustion and Aging | Castilho, Rogerio M.; Squarize, Cristiane H.; Chodosh, Lewis A.; Williams, Bart O.; Gutkind, J. Silvio | Cell Stem Cell | 2009 | 314 | 19.6 | 12 |
| 15 | Skin and Hair Follicle Integrity is Crucially Dependent on β1 Integrin Expression on Keratinocytes | Brakebusch, C; Grose, R; Quondamatteo, F; Ramirez, A; Jorcano, JL; Pirro, A; Svensson, M; Herken, R; Sasaki, T; Timpl, R; Werner, S; Fassler, R | EMBO Journal | 2000 | 304 | 12.2 | 5 |
| 16 | Role of Hormones in Pilosebaceous Unit Development | Deplewski, D; Rosenfield, RL | Endocrine Reviews | 2000 | 279 | 11.2 | 6 |
| 17 | Treatment of Hair Loss | Price, VH | New England Journal of Medicine | 1999 | 253 | 9.7 | 56 |
| 18 | Transdermal Delivery of Minoxidil with Block Copolymer Nanoparticles | Shim, J; Kang, HS; Park, WS; Han, SH; Kim, J; Chang, IS | Journal of Controlled Release | 2004 | 251 | 12.0 | 0 |
| 19 | Formulation and In Vitro Assessment of Minoxidil Niosomes for Enhanced Skin Delivery | Balakrishnana, Prabagar; Shanmugam, Srinivasan; Lee, Won Seok; Lee, Won Mo; Kim, Jong Oh; Oh, Dong Hoon; Kim, Dae-Duk; Kim, Jung Sun; Yoo, Bong Kyu; Choi, Han-Gon; Woo, Jong Soo; Yong, Chul Soon | International Journal of Pharmaceutics | 2009 | 245 | 15.3 | 9 |
| 20 | Molecular Control of Epithelial-Mesenchymal Interactions During Hair Follicle Cycling | Botchkarev, VA; Kishimoto, J | Journal of Investigative Dermatology Symposium Proceedings | 2003 | 245 | 11.1 | 10 |
| 21 | Sexual Hormones in Human Skin | Zouboulis, CC; Chen, WC; Thornton, MJ; Qin, K; Rosenfield, R | Hormone and Metabolic Research | 2007 | 244 | 13.6 | 81 |
| 22 | Diagnostic and Predictive Value of Horizontal Sections of Scalp Biopsy Specimens in Male Pattern Androgenetic Alopecia | Whiting, DA | Journal of the American Academy of Dermatology | 1993 | 243 | 7.6 | 6 |
| 23 | Localization of Androgen Receptors in Human Skin by Immunohistochemistry - Implications for the Hormonal Regulation of Hair Growth, Sebaceous Glands, and Sweat Glands | Choudhry, R; Hodgins, MB; Vanderkwast, TH; Brinkmann, AO; Boersma, WJA | Journal of Endocrinology | 1992 | 242 | 7.3 | 1 |
| 24 | WNT Signaling in the Control of Hair Growth and Structure | Millar, SE; Willert, K; Salinas, PC; Roelink, H; Nusse, R; Sussman, DJ; Barsh, GS | Developmental Biology | 1999 | 241 | 9.3 | 9 |
| 25 | Fgf9 From Dermal γδ T Cells Induces Hair Follicle Neogenesis After Wounding | Gay, Denise; Kwon, Ohsang; Zhang, Zhikun; Spata, Michelle; Plikus, Maksim V.; Holler, Phillip D.; Ito, Mayumi; Yang, Zaixin; Treffeisen, Elsa; Kim, Chang D.; Nace, Arben; Zhang, Xiaohong; Baratono, Sheena; Wang, Fen; Ornitz, David M.; Millar, Sarah E.; Cotsarelis, George | Nature Medicine | 2013 | 230 | 19.2 | 80 |
| 26 | A Review of Platelet-Rich Plasma: History, Biology, Mechanism of Action, and Classification | Alves, Rubina; Grimalt, Ramon | Skin Appendage Disorders | 2018 | 229 | 32.7 | 20 |
| 27 | In Search of the Hair Cycle Clock: A Guided Tour | Paus, R; Foitzik, K | Differentiation | 2004 | 226 | 10.8 | 7 |
| 28 | Human Skin is a Steroidogenic Tissue: Steroidogenic Enzymes and Cofactors are Expressed in Epidermis, Normal Sebocytes, and an Immortalized Sebocyte Cell Line (SEB-1) | Thiboutot, D; Jabara, S; McAllister, JM; Sivarajah, A; Gilliland, K; Cong, ZY; Clawson, G | Journal of Investigative Dermatology | 2003 | 224 | 10.2 | 4 |
| 29 | Bald Scalp in Men with Androgenetic Alopecia Retains Hair Follicle Stem Cells but Lacks CD200-Rich and CD34-Positive Hair Follicle Progenitor Cells | Garza, Luis A.; Yang, Chao-Chun; Zhao, Tailun; Blatt, Hanz B.; Lee, Michelle; He, Helen; Stanton, David C.; Carrasco, Lee; Spiegel, Jeffrey H.; Tobias, John W.; Cotsarelis, George | Journal of Clinical Investigation | 2011 | 223 | 15.9 | 50 |
| 30 | Evidence for a Single Gene Effect Causing Polycystic Ovaries and Male Pattern Baldness | Carey, AH; Chan, KL; Short, F; White, D; Williamson, R; Franks, S | Clinical Endocrinology | 1993 | 223 | 7.0 | 7 |
| 31 | A Randomized Clinical Trial of 5% Topical Minoxidil Versus 2% Topical Minoxidil and Placebo in the Treatment of Androgenetic Alopecia in Men | Olsen, EA; Dunlap, FE; Funicella, T; Koperski, JA; Swinehart, JM; Tschen, EH; Trancik, RJ | Journal of the American Academy of Dermatology | 2002 | 216 | 9.4 | 124 |
| 32 | Androgenetic Alopecia: A Review | Lolli, Francesca; Pallotti, Francesco; Rossi, Alfredo; Fortuna, Maria C.; Caro, Gemma; Lenzi, Andrea; Sansone, Andrea; Lombardo, Francesco | Endocrine | 2017 | 214 | 26.8 | 26 |
| 33 | The Effect of Platelet-Rich Plasma in Hair Regrowth: A Randomized Placebo-Controlled Trial | Gentile, Pietro; Garcovich, Simone; Bielli, Alessandra; Scioli, Maria Giovanna; Orlandi, Augusto; Cervelli, Valerio | Stem Cells Translational Medicine | 2015 | 213 | 21.3 | 490 |
| 34 | Videodermoscopy in the Evaluation of Hair and Scalp Disorders | Ross, Elizabeth K.; Vincenzi, Columbina; Tosti, Antonella | Journal of the American Academy of Dermatology | 2006 | 212 | 11.2 | 9 |
| 35 | Hair Follicle Stem Cells Provide a Functional Niche for Melanocyte Stem Cells | Tanimura, Shintaro; Tadokoro, Yuko; Inomata, Ken; Nguyen Thanh Binh; Nishie, Wataru; Yamazaki, Satoshi; Nakauchi, Hiromitsu; Tanaka, Yoshio; McMillan, James R.; Sawamura, Daisuke; Yancey, Kim; Shimizu, Hiroshi; Nishimura, Emi K. | Cell Stem Cell | 2011 | 209 | 14.9 | 8 |
| 36 | Autologous Platelet-Rich Plasma: A Potential Therapeutic Tool for Promoting Hair Growth | Li, Zheng Jun; Choi, Hye-In; Choi, Dae-Kyoung; Sohn, Kyung-Cheol; Im, Myung; Seo, Young-Joon; Lee, Young-Ho; Lee, Jeung-Hoon; Lee, Young | Dermatologic Surgery | 2012 | 207 | 15.9 | 18 |
| 37 | Localization of Androgen and Estrogen Receptors in Rat and Primate Tissues | Pelletier, G | Histology and Histopathology | 2000 | 207 | 8.3 | 0 |
| 38 | The Effectiveness of Treatments for Androgenetic Alopecia: A Systematic Review and Meta-Analysis | Adil, Areej; Godwin, Marshall | Journal of the American Academy of Dermatology | 2017 | 204 | 25.5 | 240 |
| 39 | Dermoscopy in General Dermatology: A Practical Overview | Errichetti, Enzo; Stinco, Giuseppe | Dermatology and Therapy | 2016 | 199 | 22.1 | 11 |
| 40 | The Importance of Dual 5α-Reductase Inhibition in the Treatment of Male Pattern Hair Loss: Results of a Randomized Placebo-Controlled Study of Dutasteride Versus Finasteride | Olsen, Elise A.; Hordinsky, Maria; Whiting, David; Stough, Dow; Hobbs, Stuart; Ellis, Melissa L.; Wilson, Timothy; Rittmaster, Roger S. | Journal of the American Academy of Dermatology | 2006 | 199 | 10.5 | 30 |
| 41 | The 5 Alpha-Reductase Isozyme Family: A Review of Basic Biology and their Role in Human Diseases | Azzouni, Faris; Godoy, Alejandro; Li, Yun; Mohler, James | Advances in Urology | 2012 | 197 | 15.2 | 11 |
| 42 | Molecular Mechanisms of Androgenetic Alopecia | Trüeb, RM | Experimental Gerontology | 2002 | 194 | 8.4 | 9 |
| 43 | Towards a Molecular Understanding of Hair Loss and its Treatment | Cotsarelis, G; Millar, SE | Trends in Molecular Medicine | 2001 | 194 | 8.1 | 13 |
| 44 | Adverse Side Effects of 5α-Reductase Inhibitors Therapy: Persistent Diminished Libido and Erectile Dysfunction and Depression in a Subset of Patients | Traish, Abdulmaged M.; Hassani, John; Guay, Andre T.; Zitzmann, Michael; Hansen, Michael L. | Journal of Sexual Medicine | 2011 | 193 | 13.8 | 80 |
| 45 | Minoxidil and its Use in Hair Disorders: A Review | Suchonwanit, Poonkiat; Thammarucha, Sasima; Leerunyakul, Kanchana | Drug Design Development and Therapy | 2019 | 192 | 32.0 | 618 |
| 46 | Androgen Receptor Antagonists (Antiandrogens): Structure-Activity Relationships | Singh, SM; Gauthier, S; Labrie, F | Current Medicinal Chemistry | 2000 | 191 | 7.6 | 13 |
| 47 | Dermal Papilla Cell Number Specifies Hair Size, Shape and Cycling and its Reduction Causes Follicular Decline | Chi, Woo; Wu, Eleanor; Morgan, Bruce A. | Development | 2013 | 190 | 15.8 | 1 |
| 48 | Cutaneous Androgen Metabolism: Basic Research and Clinical Perspectives | Chen, WC; Thiboutot, D; Zouboulis, CC | Journal of Investigative Dermatology | 2002 | 190 | 8.3 | 12 |
| 49 | A Guide to Studying Human Hair Follicle Cycling In Vivo | Oh, Ji Won; Kloepper, Jennifer; Langan, Ewan A.; Kim, Yongsoo; Yeo, Joongyeub; Kim, Min Ji; Hsi, Tsai-Ching; Rose, Christian; Yoon, Ghil Suk; Lee, Seok-Jong; Seykora, John; Kim, Jung Chul; Sung, Young Kwan; Kim, Moonkyu; Paus, Ralf; Plikus, Maksim V. | Journal of Investigative Dermatology | 2016 | 188 | 20.9 | 0 |
| 50 | Androgens and Hair Growth | Randall, Valerie Anne | Dermatologic Therapy | 2008 | 186 | 10.9 | 121 |
| 51 | Androgens and Male Physiology: The Syndrome of 5α-Reductase-2 Deficiency | Imperato-McGinley, J; Zhu, YS | Molecular and Cellular Endocrinology | 2002 | 186 | 8.1 | 6 |
| 52 | Hair and Scalp Dermatoscopy | Miteva, Mariya; Tosti, Antonella | Journal of the American Academy of Dermatology | 2012 | 185 | 14.2 | 3 |
| 53 | Polymorphism of the Androgen Receptor Gene is Associated with Male Pattern Baldness | Ellis, JA; Stebbing, M; Harrap, SB | Journal of Investigative Dermatology | 2001 | 185 | 7.7 | 41 |
| 54 | A Therapeutic Microneedle Patch Made from Hair-Derived Keratin for Promoting Hair Regrowth | Yang, Guang; Chen, Qian; Wen, Di; Chen, Zhaowei; Wang, Jinqiang; Chen, Guojun; Wang, Zejun; Zhang, Xudong; Zhang, Yuqi; Hu, Quanyin; Zhang, Liang; Gu, Zhen | ACS Nano | 2019 | 184 | 30.7 | 33 |
| 55 | Balding Hair Follicle Dermal Papilla Cells Contain Higher Levels of Androgen Receptors than those from Non-Balding Scalp | Hibberts, NA; Howell, AE; Randall, VA | Journal of Endocrinology | 1998 | 183 | 6.8 | 82 |
| 56 | Microneedles in the Clinic | Bhatnagar, Shubhmita; Dave, Kaushalkumar; Venuganti, Venkata Vamsi Krishna | Journal of Controlled Release | 2017 | 181 | 22.6 | 4 |
| 57 | Minoxidil Upregulates the Expression of Vascular Endothelial Growth Factor in Human Hair Dermal Papilla Cells | Lachgar, S; Charveron, M; Gall, Y; Bonafe, JL | British Journal of Dermatology | 1998 | 181 | 6.7 | 13 |
| 58 | The Biological Actions of Estrogens on Skin | Thornton, MJ | Experimental Dermatology | 2002 | 180 | 7.8 | 3 |
| 59 | Structure-Activity Relationships for Inhibition of Human 5α-Reductases by Polyphenols | Hiipakka, RA; Zhang, HZ; Dai, W; Dai, Q; Liao, ST | Biochemical Pharmacology | 2002 | 179 | 7.8 | 21 |
| 60 | Lack of Efficacy of Finasteride in Postmenopausal Women with Androgenetic Alopecia | Price, VH; Roberts, JL; Hordinsky, M; Olsen, EA; Savin, R; Bergfeld, W; Fiedler, V; Lucky, A; Whiting, DA; Pappas, F; Culbertson, J; Kotey, P; Meehan, A; Waldstreicher, J | Journal of the American Academy of Dermatology | 2000 | 178 | 7.1 | 17 |
| 61 | Psychological Effects of Androgenetic Alopecia on Women: Comparisons With Balding Men and With Female Control Subjects | Cash, TF; Price, VH; Savin, RC | Journal of the American Academy of Dermatology | 1993 | 177 | 5.5 | 14 |
| 62 | Hair Density, Hair Diameter, and the Prevalence of Female Pattern Hair Loss | Birch, MP; Messenger, JF; Messenger, AG | British Journal of Dermatology | 2001 | 176 | 7.3 | 16 |
| 63 | Differences in Hair Follicle Dermal Papilla Volume are due to Extracellular Matrix Volume and Cell Number: Implications for the Control of Hair Follicle Size and Androgen Responses | Elliott, K; Stephenson, TJ; Messenger, AG | Journal of Investigative Dermatology | 1999 | 173 | 6.7 | 10 |
| 64 | Persistent Sexual Side Effects of Finasteride for Male Pattern Hair Loss | Irwig, Michael S.; Kolukula, Swapna | Journal of Sexual Medicine | 2011 | 171 | 12.2 | 64 |
| 65 | Medical Treatments for Male and Female Pattern Hair Loss | Rogers, Nicole E.; Avram, Marc R. | Journal of the American Academy of Dermatology | 2008 | 170 | 10.0 | 54 |
| 66 | The Effect of Finasteride, a 5-Alpha-Reductase Inhibitor, on Scalp Skin Testosterone and Dihydrotestosterone Concentrations in Patients  with Male Pattern Baldness | Dallob, AL; Sadick, NS; Unger, W; Lipert, S; Geissler, LA; Gregoire, SL; Nguyen, HH; Moore, EC; Tanaka, WK | Journal of Clinical Endocrinology & Metabolism | 1994 | 166 | 5.4 | 1 |
| 67 | The CAG Repeat Polymorphism within the Androgen Receptor Gene and Maleness | Zitzmann, M; Nieschlag, E | International Journal of Andrology | 2003 | 165 | 7.5 | 3 |
| 68 | Sequence-Analysis and Expression of the cDNA for the Phenol-Sulfating Form of Human Liver Phenol Sulfotransferase | Wilborn, TW; Comer, KA; Dooley, TP; Reardon, IM; Heinrikson, RL; Falany, CN | Molecular Pharmacology | 1993 | 164 | 5.1 | 3 |
| 69 | Activating Wnt/β-Catenin Signaling Pathway for Disease Therapy: Challenges and Opportunities | Huang, Piao; Yan, Rong; Zhang, Xue; Wang, Lei; Ke, Xisong; Qu, Yi | Pharmacology & Therapeutics | 2019 | 163 | 27.2 | 1 |
| 70 | Dihydrotestosterone-Inducible Dickkopf 1 From Balding Dermal Papilla Cells Causes Apoptosis in Follicular Keratinocytes | Kwack, Mi Hee; Sung, Young Kwan; Chung, Eun Jung; Im, Sang Uk; Ahn, Ji Seop; Kim, Moon Kyu; Kim, Jung Chul | Journal of Investigative Dermatology | 2008 | 163 | 9.6 | 4 |
| 71 | A Randomized, Placebo-Controlled Trial of 5% and 2% Topical Minoxidil Solutions in the Treatment of Female Pattern Hair Loss | Lucky, AW; Piacquadio, DJ; Ditre, CM; Dunlap, F; Kantor, I; Pandya, AG; Savin, RC; Tharp, MD | Journal of the American Academy of Dermatology | 2004 | 163 | 7.8 | 71 |
| 72 | Activity of the Type-1 5-Alpha-Reductase Exhibits Regional Differences in Isolated Sebaceous Glands and Whole Skin | Thiboutot, D; Harris, G; Iles, V; Cimis, G; Gilliland, K; Hagari, S | Journal of Investigative Dermatology | 1995 | 163 | 5.4 | 3 |
| 73 | Minoxidil Use in Dermatology, Side Effects and Recent Patents | Rossi, Alfredo; Cantisani, Carmen; Melis, Luca; Iorio, Alessandra; Scali, Elisabetta; Calvieri, Stefano | Recent Patents on Inflammation & Allergy Drug Discovery | 2012 | 161 | 12.4 | 142 |
| 74 | Biology of the Hair Follicle: The Basics | Krause, K; Foitzik, K | Seminars in Cutaneous Medicine and Surgery | 2006 | 161 | 8.5 | 12 |
| 75 | The Psychosocial Consequences of Androgenetic Alopecia: A Review of the Research Literature | Cash, TF | British Journal of Dermatology | 1999 | 161 | 6.2 | 45 |
| 76 | The Role of Platelet Plasma Growth Factors in Male Pattern Baldness Surgery | Uebel, Carlos Oscar; da Silva, Jefferson Braga; Cantarelli, Denise; Martins, Pedro | Plastic and Reconstructive Surgery | 2006 | 159 | 8.4 | 3 |
| 77 | Advances in Regenerative Stem Cell Therapy in Androgenic Alopecia and Hair Loss: Wnt Pathway,  Growth-Factor, and Mesenchymal Stem Cell Signaling Impact Analysis on Cell Growth and Hair Follicle Development | Gentile, Pietro; Garcovich, Simone | Cells | 2019 | 158 | 26.3 | 15 |
| 78 | Role of TGF-β2 in the Human Hair Cycle | Hibino, T; Nishiyama, T | Journal of Dermatological Science | 2004 | 157 | 7.5 | 7 |
| 79 | The Psychological Effects of Androgenetic Alopecia in Men | Cash, TF | Journal of the American Academy of Dermatology | 1992 | 157 | 4.8 | 22 |
| 80 | Prevalence of Male and Female Pattern Hair Loss in Maryborough | Gan, DCC; Sinclair, RD | Journal of Investigative Dermatology Symposium Proceedings | 2005 | 156 | 7.8 | 128 |
| 81 | Female Pattern Hair Loss | Olsen, EA | Journal of the American Academy of Dermatology | 2001 | 156 | 6.5 | 0 |
| 82 | The Effect of Hair Loss on Quality of Life | Williamson, D; Gonzalez, M; Finlay, AY | Journal of the European Academy of Dermatology and Venereology | 2001 | 155 | 6.5 | 12 |
| 83 | Hair Follicle Dermal Stem Cells Regenerate the Dermal Sheath, Repopulate the Dermal Papilla, and Modulate Hair Type | Rahmani, Waleed; Abbasi, Sepideh; Hagner, Andrew; Raharjo, Eko; Kumar, Ranjan; Hotta, Akitsu; Magness, Scott; Metzger, Daniel; Biernaskie, Jeff | Developmental Cell | 2014 | 153 | 13.9 | 19 |
| 84 | Androgen-Inducible TGF-β1 From Balding Dermal Papilla Cells Inhibits Epithelial Cell Growth: A Clue to Understanding Paradoxical Effects of Androgen on Human Hair Growth | Inui, S; Fukuzato, Y; Nakajima, T; Yoshikawa, K; Itami, S | FASEB Journal | 2002 | 153 | 6.7 | 3 |
| 85 | Evidence-Based (S3) Guideline for the Treatment of Androgenetic Alopecia in Women and in Men | Blumeyer, Anja; Tosti, Antonella; Messenger, Andrew; Reygagne, Pascal; del Marmol, Veronique; Spuls, Phyllis I.; Trakatelli, Myrto; Finner, Andreas; Kiesewetter, Franklin; Trueeb, Ralph; Rzany, Berthold; Blume-Peytavi, Ulrike | Journal der Deutschen Dermatologischen Gesellschaft | 2011 | 152 | 10.9 | 33 |
| 86 | Evaluation and Treatment of Male and Female Pattern Hair Loss | Olsen, EA; Messenger, AG; Shapiro, J; Bergfeld, WF; Hordinsky, MK; Roberts, JL; Stough, D; Washenik, K; Whiting, DA | Journal of the American Academy of Dermatology | 2005 | 150 | 7.5 | 16 |
| 87 | Genetic Variation in the Human Androgen Receptor Gene is the Major Determinant of Common Early-Onset Androgenetic Alopecia | Hillmer, AM; Hanneken, S; Ritzmann, S; Becker, T; Freudenberg, J; Brockschmidt, FF; Flaquer, A; Freudenberg-Hua, Y; Abou Jamra, R; Metzen, C; Heyn, U; Schweiger, N; Betz, RC; Blaumeiser, B; Hampe, J; Schreiber, S; Schulze, TG; Hennies, HC; Schumacher, J; Propping, P; Ruzicka, T; Cichon, S; Wienker, TF; Kruse, R; Nöthen, MM | American Journal of Human Genetics | 2005 | 148 | 7.4 | 60 |
| 88 | Androgenetic Alopecia in Men Aged 40-69 Years: Prevalence and Risk Factors | Severi, G; Sinclair, R; Hopper, JL; English, DR; McCredie, MRE; Boyle, P; Giles, GG | British Journal of Dermatology | 2003 | 148 | 6.7 | 6 |
| 89 | Minoxidil Sulfate is the Active Metabolite that Stimulates Hair Follicles | Buhl, AE; Waldon, DJ; Baker, CA; Johnson, GA | Journal of Investigative Dermatology | 1990 | 147 | 4.2 | 10 |
| 90 | Trichoscopy Update 2011 | Rudnicka, Lidia; Olszewska, Malgorzata; Rakowska, Adriana; Slowinska, Monika | Journal of Dermatological Case Reports | 2011 | 146 | 10.4 | 1 |
| 91 | Current and Novel Methods for Assessing Efficacy of Hair Growth Promoters in Pattern Hair Loss | Olsen, EA | Journal of the American Academy of Dermatology | 2003 | 145 | 6.6 | 6 |
| 92 | Pharmacologic Inhibition of JAK-STAT Signaling Promotes Hair Growth | Harel, Sivan; Higgins, Claire A.; Cerise, Jane E.; Dai, Zhenpeng; Chen, James C.; Clynes, Raphael; Christiano, Angela M. | Science Advances | 2015 | 144 | 14.4 | 441 |
| 93 | The Effect of Autologous Activated Platelet-Rich Plasma (AA-PRP) Injection on Pattern Hair Loss: Clinical and Histomorphometric Evaluation | Cervelli, V.; Garcovich, S.; Bielli, A.; Cervelli, G.; Curcio, B. C.; Scioli, M. G.; Orlandi, A.; Gentile, P. | Biomed Research International | 2014 | 144 | 13.1 | 17 |
| 94 | Effects of Sex Steroid Deprivation/Administration on Hair Growth and Skin Sebum Production in Transsexual Males and Females | Giltay, EJ; Gooren, LJG | Journal of Clinical Endocrinology & Metabolism | 2000 | 144 | 5.8 | 7 |
| 95 | Penetration of Laser Light at 808 and 980 nm in Bovine Tissue Samples | Hudson, Donald E.; Hudson, Doreen O.; Wininger, James M.; Richardson, Brian D. | Photomedicine and Laser Surgery | 2013 | 142 | 11.8 | 3 |
| 96 | Effect of Minoxidil on Proliferation and Apoptosis in Dermal Papilla Cells of Human Hair Follicle | Han, JH; Kwon, OS; Chung, JH; Cho, KH; Eun, HC; Kim, KH | Journal of Dermatological Science | 2004 | 142 | 6.8 | 9 |
| 97 | Burden of Hair Loss: Stress and the Underestimated Psychosocial Impact of Telogen Effluvium and Androgenetic Alopecia | Hadshiew, IM; Foitzik, K; Arck, PC; Paus, R | Journal of Investigative Dermatology | 2004 | 141 | 6.7 | 81 |
| 98 | Finasteride in the Treatment of Men with Frontal Male Pattern Hair Loss | Leyden, J; Dunlap, F; Miller, B; Winters, P; Lebwohl, M; Hecker, D; Kraus, S; Baldwin, H; Shalita, A; Draelos, Z; Markou, M; Thiboutot, D; Rapaport, M; Kang, SW; Kelly, T; Pariser, D; Webster, G; Hordinsky, M; Rietschel, R; Katz, HI; Terranella, L; Best, S; Round, E; Waldstreicher, J | Journal of the American Academy of Dermatology | 1999 | 141 | 5.4 | 9 |
| 99 | Clinical and Biochemical Parameters of Androgen Action in Normal Healthy Caucasian versus Chinese Subjects | Lookingbill, DP; Demers, LM; Wang, C; Leung, A; Rittmaster, RS; Santen, RJ | Journal of Clinical Endocrinology & Metabolism | 1991 | 141 | 4.2 | 14 |
| 100 | Gender Differences in Skin: A Review of the Literature | Dao, Harry, Jr.; Kazin, Rebecca A. | Gender Medicine | 2007 | 140 | 7.8 | 11 |

**Table S1.** List of Top 100 Most Cited AGA Articles.
