# Supplementary material for: The 100 most cited articles in androgenetic alopecia: A bibliometric analysis
Source: Medicine (Baltimore). 2025 Mar 21;104(12):e41881. doi: 10.1097/MD.0000000000041881 (PMC11936583; doi:10.1097/MD.0000000000041881)
Supplement: SUPPLEMENTARY MATERIAL [file medi-104-e41881-s002.docx]

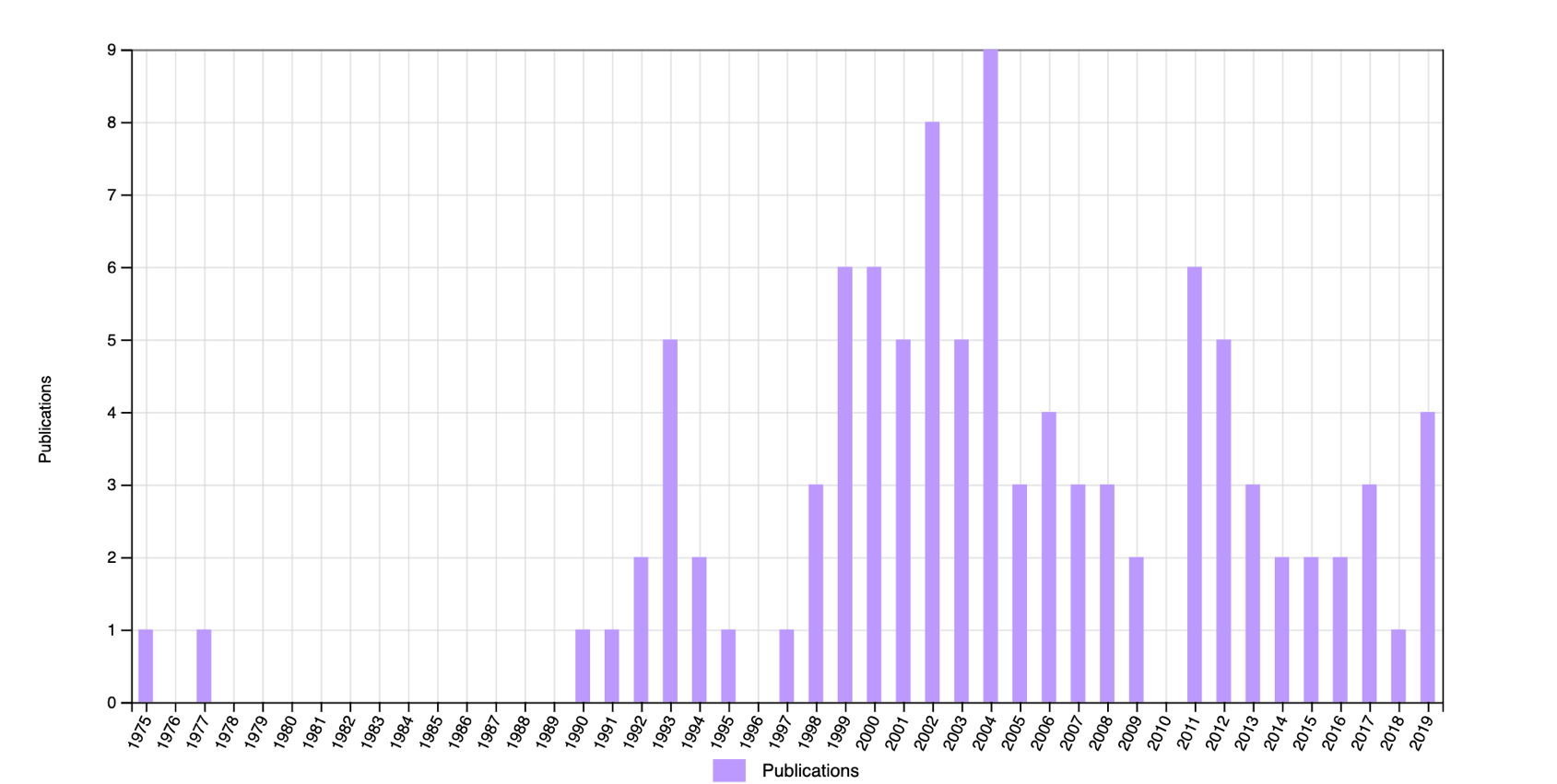


**Figure S1.** Total publications per year for the top 100 cited articles in AGA between the years 1975 and 2024.


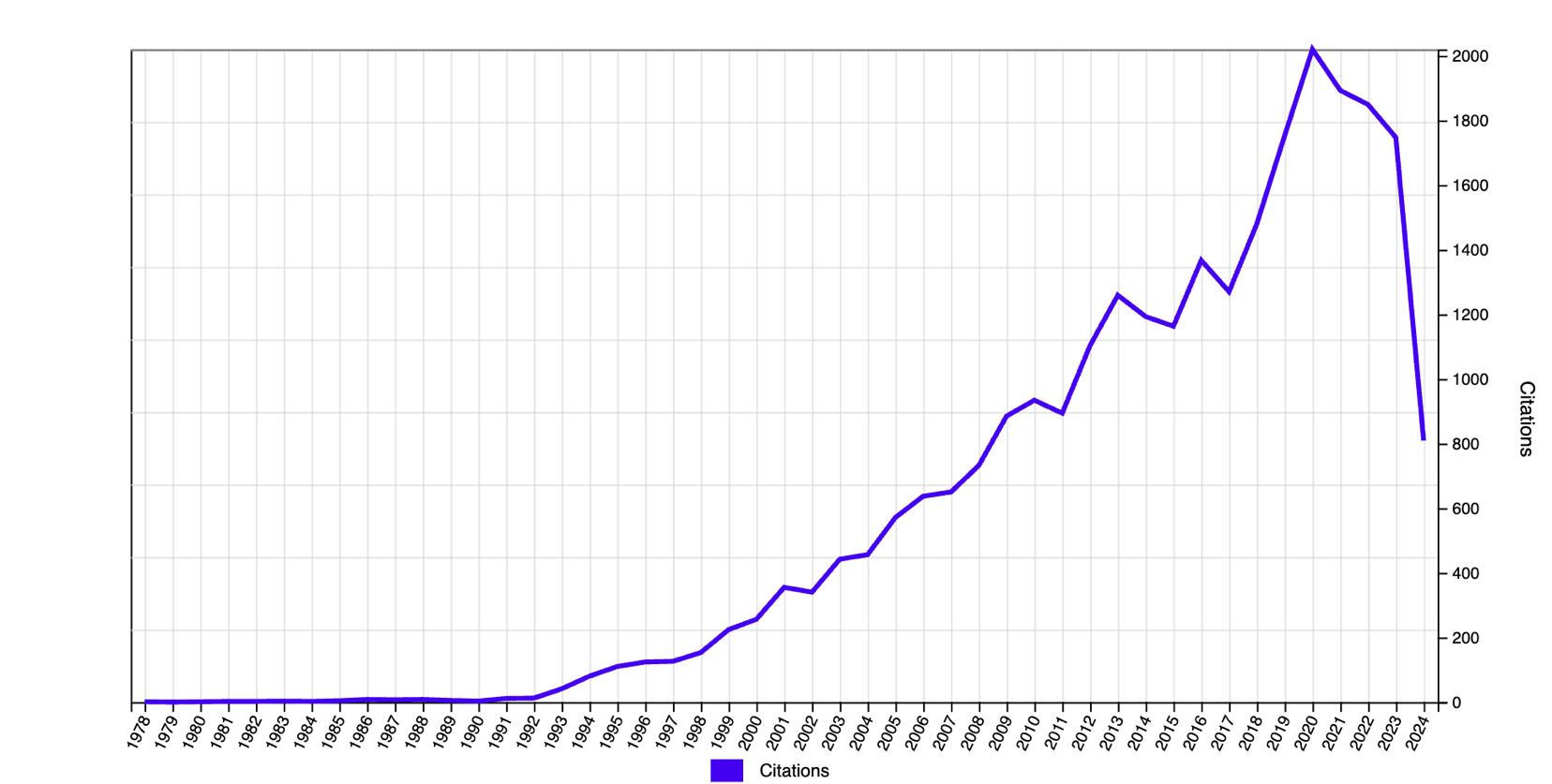


**Figure S2.** Number of citations per year for the top 100 cited articles in AGA.
