# Supplementary material for: The 100 most cited articles in androgenetic alopecia: A bibliometric analysis
Source: Medicine (Baltimore). 2025 Mar 21;104(12):e41881. doi: 10.1097/MD.0000000000041881 (PMC11936583; doi:10.1097/MD.0000000000041881)
Supplement: SUPPLEMENTARY MATERIAL [file medi-104-e41881-s003.docx]

| **Rank** | **Countries** | **Top 100 Articles*** |
| --- | --- | --- |
| 1 | USA | 56 |
| 2 | England | 14 |
| 3 | Germany | 12 |
| 4 | Canada | 9 |
| 5 | Italy | 9 |
| 6 | UK | 8 |
| 7 | South Korea | 7 |
| 8 | Japan | 5 |
| 9 | Belgium | 4 |
| 10 | Netherlands | 4 |
| 11 | Australia | 3 |
| 12 | France | 3 |
| 13 | People’s Republic of China | 3 |
| 14 | Switzerland | 3 |
| 15 | Taiwan | 3 |
| 16 | Spain | 2 |
| 17 | Sweden | 2 |

**Table S2.** Countries contributing to the top 100 list.

*Multiple articles had contributions by >1 country.
