# Supplementary material for: The 100 most cited articles in androgenetic alopecia: A bibliometric analysis
Source: Medicine (Baltimore). 2025 Mar 21;104(12):e41881. doi: 10.1097/MD.0000000000041881 (PMC11936583; doi:10.1097/MD.0000000000041881)
Supplement: SUPPLEMENTARY MATERIAL [file medi-104-e41881-s004.docx]

| Rank | Organization* | Top 100 Articles |
| --- | --- | --- |
| 1 | University of Pennsylvania | 11 |
| 2 | Duke University | 7 |
| 3 | University of California System | 7 |
| 4 | University of Texas System | 7 |
| 5 | Pennsylvania Commonwealth System Of Higher Education | 6 |
| 6 | Merck Company | 5 |
| 7 | Penn State Health | 5 |
| 8 | Pennsylvania State University | 5 |
| 9 | Pfizer | 5 |
| 10 | University Of Hamburg | 5 |
| 11 | University Of Sheffield | 5 |
| 12 | Charite Universitatsmedizin Berlin | 4 |
| 13 | Free University of Berlin | 4 |
| 14 | Humboldt University of Berlin | 4 |
| 15 | University of Bradford | 4 |
| 16 | University of California San Francisco | 4 |
| 17 | University of Chicago | 4 |
| 18 | University of Minnesota System | 4 |
| 19 | University of Minnesota Twin Cities | 4 |
| 20 | University of Texas Dallas | 4 |
| 21 | University of Texas Southwestern Medical Center Dallas | 4 |
| 22 | Argus Research Inc | 3 |
| 23 | Baylor Hair Research Treatment Center | 3 |
| 24 | Boston University | 3 |
| 25 | Catholic University of The Sacred Heart | 3 |
| 26 | Cleveland Clinic Foundation | 3 |
| 27 | Imperial College London | 3 |
| 28 | Irccs Policlinico Gemelli | 3 |
| 29 | Old Dominion University | 3 |
| 30 | Seoul National University | 3 |
| 31 | University of Melbourne | 3 |
| 32 | University of Munster | 3 |
| 33 | University of Rome Tor Vergata | 3 |

**Table S3.** Organizations contributing to the top 100 list.

*195 additional organizations that contributed to **≤**2 publications were excluded. Additionally, multiple articles were associated with >1 contributing institution.
