# Supplementary material for: The 100 most cited articles in androgenetic alopecia: A bibliometric analysis
Source: Medicine (Baltimore). 2025 Mar 21;104(12):e41881. doi: 10.1097/MD.0000000000041881 (PMC11936583; doi:10.1097/MD.0000000000041881)
Supplement: SUPPLEMENTARY MATERIAL [file medi-104-e41881-s005.docx]

| Rank | Funding Agencies* | Top 100 Articles |
| --- | --- | --- |
| 1 | National Institutes of Health NIH USA | 14 |
| 2 | United States Department of Health Human Services | 14 |
| 3 | NIH Eunice Kennedy Shriver National Institute of Child Health Human Development NICHD | 5 |
| 4 | NIH National Institute of Arthritis Musculoskeletal Skin Diseases NIAMS | 5 |
| 5 | Dermatology Foundation | 2 |
| 6 | Edwin and Fannie Gray Hall Center for Human Appearance at University of Pennsylvania Medical Center | 2 |
| 6 | NIH National Cancer Institute NCI | 2 |
| 7 | NIH National Center for Research Resources NCRR | 2 |
| 8 | NIH National Institute of General Medical Sciences NIGMS | 2 |
| 9 | AIHS MD PhD | 1 |
| 10 | Air Force Office of Scientific Research (AFOSR) | 1 |
| 11 | Airlift Research Foundation | 1 |
| 12 | Alberta Innovates | 1 |
| 13 | Alopecia Areata Initiative | 1 |
| 14 | American Skin Association | 1 |
| 15 | Basic Research Program of the Korean Science and Engineering Foundation in South Korea | 1 |
| 16 | Basic Science Research Program Through the National Research Foundation of Korea NRF Ministry of Education Science and Technology | 1 |
| 17 | BITS Pilani Hyderabad Campus | 1 |
| 18 | Calgary Firefighters Burn Treatment Society | 1 |
| 19 | Canadian Institutes of Health Research (CIHR) | 1 |
| 20 | Center for Integration of Medicine and Innovative Technology | 1 |
| 21 | Chinese Academy of Sciences | 1 |
| 22 | Chungnam National University | 1 |
| 23 | Craniofacial Tissue Remodeling Initiative of the NIH | 1 |
| 24 | Department of Urology Boston University School of Medicine | 1 |
| 25 | Dermatology Foundation Career Development Award | 1 |
| 26 | Edward Mallinckrodt Jr Foundation Grant | 1 |
| 27 | ETRI RD Program | 1 |
| 30 | European Dermatology Forum (EDF) | 1 |
| 31 | German Research Foundation DFG | 1 |
| 32 | Grants in Aid for Scientific Research KAKENHI | 1 |
| 33 | Intramural Research Program | 1 |
| 34 | Johnson & Johnson | 1 |
| 35 | Japan Society for the Promotion of Science | 1 |
| 36 | Kato Memorial Bioscience Foundation | 1 |
| 37 | Kirschstein National Research Service Award Postdoctoral Training Grant in Medical Genetics | 1 |
| 38 | Kyungpook National University Research Fund | 1 |
| 39 | L'Oréal Group | 1 |
| 40 | Locks of Love Foundation | 1 |
| 41 | Meda | 1 |
| 42 | Ministry of Education Culture Sports Science and Technology Japan MEXT | 1 |
| 43 | Ministry of Education Universities and Research MIUR | 1 |
| 44 | Ministry of Trade Industry Energy MOTIE Republic of Korea | 1 |
| 45 | National Key Research and Development Program of China | 1 |
| 46 | National Natural Science Foundation of China NSFC | 1 |
| 47 | NIH National Institute of Dental Craniofacial Research NIDCR | 1 |
| 48 | NIH National Institute of Diabetes Digestive Kidney Diseases NIDDK | 1 |
| 49 | NIH Skin Diseases Research Core | 1 |
| 50 | Pennsylvania Department of Health | 1 |
| 51 | Regenerative Medicine R&D Fund by Daegu City Korea | 1 |
| 52 | Science Technology Commission of Shanghai Municipality STCSM | 1 |
| 53 | Shanghai Pujiang Program | 1 |
| 54 | Stem Cell Network Global Impact Grant | 1 |
| 55 | Takeda Science Foundation (TSF) | 1 |
| 56 | U.S. Public Health Service NIH National Institute of Arthritis and Musculoskeletal and Skin Diseases | 1 |
| 57 | UCVM Dean's Excellence Award | 1 |
| 58 | Uehara Memorial Foundation | 1 |
| 59 | UNC Chapel Hill NC State | 1 |
| 60 | United States Department of Defense | 1 |
| 61 | United States Public Health Service | 1 |
| 62 | University of California Cancer Research Coordinating Committee CRCC Grant | 1 |
| 63 | University of California System | 1 |
| 64 | University of Rome La Sapienza Faculty of Medicine | 1 |

**Table S4.** Funding Agencies contributing to the top 100 list.

*Some articles received financial support from multiple funding agencies.
