# Supplementary material for: The 100 most cited articles in androgenetic alopecia: A bibliometric analysis
Source: Medicine (Baltimore). 2025 Mar 21;104(12):e41881. doi: 10.1097/MD.0000000000041881 (PMC11936583; doi:10.1097/MD.0000000000041881)
Supplement: SUPPLEMENTARY MATERIAL [file medi-104-e41881-s006.docx]

| Rank | Journal Title | Top 100 Publications | Total Number of Citations in Top 100 | Impact Factor | Eigenfactor | Article Influence |
| --- | --- | --- | --- | --- | --- | --- |
| 1 | Journal of the American Academy of Dermatology | 16 | 3161 | 12.8 | 0.037 | 3.635 |
| 2 | Journal of Investigative Dermatology | 11 | 2642 | 5.7 | 0.021 | 2.031 |
| 3 | British Journal of Dermatology | 6 | 1534 | 11.0 | 0.026 | 2.982 |
| 4 | Journal of Clinical Endocrinology Metabolism | 4 | 995 | 5.0 | 0.053 | 1.830 |
| 5 | Cell Stem Cell | 2 | 523 | 19.8 | 0.051 | 10.417 |
| 6 | Journal of Clinical Investigation | 2 | 824 | 13.3 | 0.095 | 5.435 |
| 7 | Journal of Controlled Release | 2 | 432 | 10.5 | 0.041 | 1.789 |
| 8 | Journal of Dermatological Science | 2 | 299 | 3.8 | 0.004 | 1.052 |
| 9 | Journal of Endocrinology | 2 | 425 | 3.4 | 0.005 | 1.118 |
| 10 | Journal of Investigative Dermatology Symposium Proceedings | 2 | 401 | 3.7 | 0.001 | 0.726 |
| 11 | Journal of Sexual Medicine | 2 | 364 | 3.3 | 0.008 | 0.999 |
| 12 | New England Journal of Medicine | 2 | 1151 | 96.2 | 0.561 | 41.530 |
| 13 | ACS Nano | 1 | 184 | 15.8 | 0.227 | 3.530 |
| 14 | Advances in Urology | 1 | 197 | 1.8 | 0.001 | 0.952 |
| 15 | American Journal of Human Genetics | 1 | 148 | 8.1 | 0.037 | 5.294 |
| 16 | Annals of Biomedical Engineering | 1 | 883 | 3.0 | 0.007 | 0.760 |
| 17 | Biochemical Pharmacology | 1 | 179 | 5.3 | 0.019 | 1.109 |
| 18 | Biomed Research International | 1 | 144 | 2.6 | 0.052 | 0.609 |
| 19 | Cells | 1 | 158 | 5.1 | 0.135 | 1.337 |
| 20 | Clinical Endocrinology | 1 | 223 | 3.0 | 0.009 | 1.080 |
| 21 | Current Medicinal Chemistry | 1 | 191 | 3.5 | 0.010 | 0.708 |
| 22 | Dermatologic Surgery | 1 | 207 | 2.5 | 0.006 | 0.813 |
| 23 | Dermatologic Therapy | 1 | 186 | 3.7 | 0.012 | 0.799 |
| 24 | Dermatology and Therapy | 1 | 199 | 3.5 | 0.005 | 0.997 |
| 25 | Development | 1 | 190 | 3.7 | 0.036 | 2.218 |
| 26 | Developmental Biology | 1 | 241 | 2.5 | 0.009 | 1.057 |
| 27 | Developmental Cell | 1 | 153 | 10.7 | 0.046 | 5.407 |
| 28 | Differentiation | 1 | 226 | 2.2 | 0.001 | 0.670 |
| 29 | Drug Design Development and Therapy | 1 | 192 | 4.7 | 0.013 | 0.856 |
| 30 | EMBO Journal | 1 | 304 | 9.4 | 0.047 | 4.867 |
| 31 | Endocrine | 1 | 214 | 3.0 | 0.011 | 0.812 |
| 32 | Endocrine Reviews | 1 | 279 | 22.0 | 0.015 | 8.787 |
| 33 | Experimental Dermatology | 1 | 180 | 3.5 | 0.009 | 1.075 |
| 34 | Experimental Gerontology | 1 | 194 | 3.3 | 0.011 | 0.907 |
| 35 | FASEB Journal | 1 | 153 | 4.4 | 0.043 | 1.252 |
| 36 | Gender Medicine | 1 | 140 | 2.3 | 0.002 | 0.738 |
| 37 | Histology and Histopathology | 1 | 207 | 2.5 | 0.002 | 0.409 |
| 38 | Hormone and Metabolic Research | 1 | 244 | 2.0 | 0.002 | 0.510 |
| 39 | Human Molecular Genetics | 1 | 380 | 3.1 | 0.024 | 1.692 |
| 40 | International Journal of Andrology | 1 | 165 | 3.7 | 0.006 | 0.988 |
| 41 | International Journal of Pharmaceutics | 1 | 245 | 5.3 | 0 | 0 |
| 42 | Journal der Deutschen Dermatologischen Gesellschaft | 1 | 152 | 5.5 | 0.003 | 0.985 |
| 43 | Journal of Dermatological Case Reports | 1 | 146 | * | * | * |
| 44 | Journal of the European Academy of Dermatology and Venereology | 1 | 155 | 8.4 | 0.024 | 1.941 |
| 45 | Molecular and Cellular Endocrinology | 1 | 186 | 3.8 | 0.010 | 0.921 |
| 46 | Molecular Pharmacology | 1 | 164 | 3.2 | 0.004 | 0.997 |
| 47 | Nature | 1 | 796 | 50.5 | 1.025 | 24.739 |
| 48 | Nature Biotechnology | 1 | 1019 | 33.1 | 0.156 | 27.931 |
| 49 | Nature Medicine | 1 | 230 | 58.7 | 0.227 | 25.942 |
| 50 | Pharmacology & Therapeutics | 1 | 163 | 12.0 | 0.020 | 2.919 |
| 51 | Photomedicine and Laser Surgery | 1 | 142 | 2.8 | 0.002 | 0.575 |
| 52 | Plastic and Reconstructive Surgery | 1 | 159 | 3.2 | 0.022 | 1.097 |
| 53 | Recent Patents on Inflammation & Allergy Drug Discovery | 1 | 161 | 4.2 | 0.001 | 1.053 |
| 54 | Science Advances | 1 | 144 | 11.7 | 0.355 | 5.091 |
| 55 | Seminars in Cutaneous Medicine and Surgery | 1 | 161 | 1.1 | 0.001 | 0.316 |
| 56 | Skin Appendage Disorders | 1 | 229 | 1.4 | 0.002 | 0.485 |
| 57 | Southern Medical Journal | 1 | 518 | 1.0 | 0.002 | 0.338 |
| 58 | Stem Cells Translational Medicine | 1 | 213 | 5.4 | 0.007 | 1.378 |
| 59 | Trends in Molecular Medicine ​​ | 1 | 194 | 12.8 | 0.013 | 4.033 |

**Table S5.** Journals represented in the top 100 list.

*Journal discontinued, results unavailable
