# Supplementary material for: The 100 most cited articles in androgenetic alopecia: A bibliometric analysis
Source: Medicine (Baltimore). 2025 Mar 21;104(12):e41881. doi: 10.1097/MD.0000000000041881 (PMC11936583; doi:10.1097/MD.0000000000041881)
Supplement: SUPPLEMENTARY MATERIAL [file medi-104-e41881-s007.docx]

| Rank | Title | Authors | Journal | Publication Year | Total Citations | Average per Year | Altmetric Score |
| --- | --- | --- | --- | --- | --- | --- | --- |
| 1 | Targeting Wnt/β-Catenin Pathway for Developing Therapies for Hair Loss | Choi, Bu Young | International Journal of Molecular Sciences | 2020 | 112 | 22.4 | 13 |
| 2 | Treatment options for androgenetic alopecia: Efficacy, side effects, compliance, financial considerations, and ethics | Nestor, Mark S.; Ablon, Glynis; Gade, Anita; Han, Haowei; Fischer, Daniel L. | Journal of Cosmetic Dermatology | 2021 | 101 | 25.3 | 82 |
| 3 | Dermal exosomes containing miR-218-5p promote hair regeneration by regulating β-catenin signaling | Hu, Shiqi; Li, Zhenhua; Lutz, Halle; Huang, Ke; Su, Teng; Cores, Jhon; Dinh, Phuong-Uyen Cao; Cheng, Ke | Science Advances | 2020 | 99 | 19.8 | 183 |
| 4 | Autologous Cellular Method Using Micrografts of Human Adipose Tissue Derived Follicle Stem Cells in Androgenic Alopecia | Gentile, Pietro | International Journal of Molecular Sciences | 2019 | 98 | 16.3 | 1 |
| 5 | Self-Activated Electrical Stimulation for Effective Hair Regeneration via a Wearable Omnidirectional Pulse Generator | Yao, Guang; Jiang, Dawei; Li, Jun; Kang, Lei; Chen, Sihong; Long, Yin; Wang, Yizhan; Huang, Peng; Lin, Yuan; Cai, Weibo; Wang, Xudong | ACS Nano | 2019 | 95 | 15.8 | 383 |
| 6 | Hormonal Effects on Hair Follicles | Grymowicz, Monika; Rudnicka, Ewa; Podfigurna, Agnieszka; Napierala, Paulina; Smolarczyk, Roman; Smolarczyk, Katarzyna; Meczekalski, Blazej | International Journal of Molecular Sciences | 2020 | 92 | 18.4 | 564 |
| 7 | Ceria Nanozyme-Integrated Microneedles Reshape the Perifollicular Microenvironment for Androgenetic Alopecia Treatment | Yuan, Anran; Xia, Fan; Bian, Qiong; Wu, Haibin; Gu, Yueting; Wang, Tao; Wang, Ruxuan; Huang, Lingling; Huang, Qiaoling; Rao, Yuefeng; Ling, Daishun; Li, Fangyuan; Gao, Jianqing | ACS Nano | 2021 | 89 | 22.3 | 202 |
| 8 | Platelet-rich plasma and its utility in medical dermatology: A systematic review | Hesseler, Michael J.; Shyam, Nikhil | Journal of the American Academy of Dermatology | 2019 | 89 | 14.8 | 28 |
| 9 | Rosemary (Rosmarinus officinalis L., syn Salvia Rosmarinus Spenn.) and Its Topical Applications: A Review | de Macedo, Lucas Malvezzi; dos Santos, Erica Mendes; Militao, Lucas; Tundisi, Louise Lacalendola; Ataide, Janaina Artem; Souto, Eliana Barbosa; Mazzola, Priscila Gava | Plants-Basel | 2020 | 88 | 17.6 | 50 |
| 10 | Systematic Review of Platelet-Rich Plasma Use in Androgenetic Alopecia Compared with Minoxidil, Finasteride, and Adult Stem Cell-Based Therapy | Gentile, Pietro; Garcovich, Simone | International Journal of Molecular Sciences | 2020 | 86 | 17.2 | 12 |
| 11 | Treatment review for male pattern hair-loss | York, Katherine; Meah, Nekma; Bhoyrul, Bevin; Sinclair, Rodney | Expert Opinion on Pharmacotherapy | 2020 | 82 | 16.4 | 28 |
| 12 | Ultrasound-activated particles as CRISPR/Cas9 delivery system for androgenic alopecia therapy | Ryu, Jee-Yeon; Won, Eun-Jeong; Lee, Han A. Reum; Kim, Jin Hyun; Hui, Emmanuel; Kim, Hong Pyo; Yoon, Tae-Jong | Biomaterials | 2020 | 77 | 15.4 | 0 |
| 13 | Impact of the Different Preparation Methods to Obtain Autologous Non-Activated Platelet-Rich Plasma (A-PRP) and Activated Platelet-Rich Plasma (AA-PRP) in Plastic Surgery: Wound Healing and Hair Regrowth Evaluation | Gentile, Pietro; Calabrese, Claudio; De Angelis, Barbara; Dionisi, Laura; Pizzicannella, Jacopo; Kothari, Ashutosh; De Fazio, Domenico; Garcovich, Simone | International Journal of Molecular Sciences | 2020 | 77 | 15.4 | 0 |
| 14 | Minoxidil: a comprehensive review | Gupta, A. K.; Talukder, M.; Venkataraman, M.; Bamimore, M. A. | Journal of Dermatological Treatment | 2022 | 72 | 18.0 | 176 |
| 15 | Applications of the regenerative capacity of platelets in modern medicine | Cecerska-Heryc, Elibieta; Goszka, Malgorzata; Serwin, Natalia; Roszak, Marta; Grygorcewicz, Bartlomiej; Heryc, Rafal; Dolegowska, Barbara | Cytokine & Growth Factor Reviews | 2022 | 71 | 23.7 | 13 |
| 16 | Exosomes derived from human dermal papilla cells promote hair growth in cultured human hair follicles and augment the hair-inductive capacity of cultured dermal papilla spheres | Kwack, Mi H.; Seo, Chang H.; Gangadaran, Prakash; Ahn, Byeong-Cheol; Kim, Moon K.; Kim, Jung C.; Sung, Young K. | Experimental Dermatology | 2019 | 71 | 11.8 | 4 |
| 17 | Functional complexity of hair follicle stem cell niche and therapeutic targeting of niche dysfunction for hair regeneration | Chen, Chih-Lung; Huang, Wen-Yen; Wang, Eddy Hsi Chun; Tai, Kang-Yu; Lin, Sung-Jan | Journal of Biomedical Science | 2020 | 68 | 13.6 | 4 |
| 18 | Minoxidil 1 mg oral versus minoxidil 5% topical solution for the treatment of female-pattern hair loss: A randomized clinical trial | Ramos, Paulo Mueller; Sinclair, Rodney D.; Kasprzak, Michal; Miot, Helio Amante | Journal of the American Academy of Dermatology | 2020 | 68 | 13.6 | 42 |
| 19 | Dihydrotestosterone-induced hair regrowth inhibition by activating androgen receptor in C57BL6 mice simulates androgenetic alopecia | Fu, Danlan; Huang, Junfei; Li, Kaitao; Chen, Yuxin; He, Ye; Sun, Yang; Guo, Yilong; Du, Lijuan; Qu, Qian; Miao, Yong; Hu, Zhiqi | Biomedicine & Pharmacotherapy | 2021 | 67 | 16.8 | 1 |
| 20 | Female Androgenetic Alopecia: An Update on Diagnosis and Management | Starace, Michela; Orlando, Gloria; Alessandrini, Aurora; Piraccini, Bianca Maria | American Journal of Clinical Dermatology | 2020 | 66 | 11.0 | 33 |

**Table S6.** Top 20 most cited articles related to AGA from 2020-2024.
